# A 50 kdyne contusion spinal cord injury with or without the drug SS‐31 was not associated with major changes in muscle mass or gene expression 14 d after injury in young male mice

**DOI:** 10.14814/phy2.14751

**Published:** 2021-02-21

**Authors:** Zachary A. Graham, Jennifer J. DeBerry, Christopher P. Cardozo, Marcas M. Bamman

**Affiliations:** ^1^ Research Service Birmingham VA Medical Center Birmingham AL USA; ^2^ Department of Cell, Developmental, and Integrative Biology University of Alabama‐Birmingham Birmingham AL USA; ^3^ Department of Anesthesiology and Perioperative Medicine University of Alabama‐Birmingham Birmingham AL USA; ^4^ National Center for the Medical Consequences of Spinal Cord Injury, James J. Peters VA Medical Center Bronx NY USA; ^5^ Medical Service James J. Peters VA Medical Center Bronx NY USA; ^6^ Departments of Medicine and Rehabilitation Medicine Icahn School of Medicine at Mount Sinai New York NY USA; ^7^ UAB Center for Exercise Medicine University of Alabama‐Birmingham Birmingham AL USA

**Keywords:** paralysis, skeletal muscle, spinal cord injury, SS‐31

## Abstract

Spinal cord injury (SCI) leads to rapid muscle atrophy due to paralysis/paresis and subsequent disuse. SS‐31 is a mitochondrial‐targeting peptide that has shown efficacy in protecting skeletal muscle mass and function in non‐SCI models of muscle wasting. We aimed to determine if SS‐31 could prevent muscle loss after SCI. Male C57BL/6 mice aged 9 weeks underwent sham surgery or 50 kdyne contusion SCI and were administered daily injections of vehicle or 5 mg/kg SS‐31 for 14 d. Both SCI groups had sustained losses in body mass compared to Sham animals and ~10% reductions in gastrocnemius, plantaris and tibialis anterior muscle mass after SCI with no clear effect of SS‐31. Measurements of protein synthesis in the soleus and plantaris were similar among all groups. mRNA expression of atrophy‐associated proinflammatory cytokines was also similar among all groups. There was elevation in *MYH7* mRNA and a statistical reduction in *MYH2* mRNA expression in the SCI+SS‐31 animals compared to Sham animals. There was an SCI‐induced reduction in mRNA expression of the E3 ligase *FBXO32* (MAFbx), but no effect of SS‐31. In summary, a 50 kdyne contusion SCI was able to reduce body mass but was not associated with substantial muscle atrophy or alterations in gene expression profiles associated with muscle health and function 14 d post‐injury. SS‐31 was not associated with protection against SCI‐related changes in body or muscle mass, protein synthesis or gene expression in hindlimb muscles.

## Graphical abstract

SS‐31 is an efficacious treatment for many pre‐clinical models of muscle disuse. We show that daily injections of SS‐31 were not able to prevent muscle or body mass losses 14 d after an acute contusion SCI in mice.

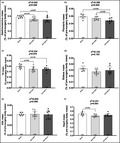

## 1 INTRODUCTION

Spinal cord injury (SCI) affects roughly 290,000 people living in the USA with a majority of injuries from 2015–18 being neurologically incomplete injuries (National Spinal Cord Injury Statistical Center, 2020). Complete anatomical spinal cord transections are rare in those with SCI but result in functionally complete motor and/or sensory injuries. The preservation of myelinated axons that transverse the glial scar in most cases of SCI allow for some capacity of sensation and motor recovery. Neurological improvements are negligible (but possible) for those with SCI with a grade of American Spinal Injury Association (ASIA) A (motor and sensory complete) from 1 y to 5 y post‐injury (Kirshblum et al., 2004). Those with less severe ASIA B‐D injuries show greater capacity to recover both between discharge and 1 y from the date of injury (Vazquez et al., 2008) as well as 1 y to 5 y post‐injury (Kirshblum et al., 2004). Functional recovery using measures of standing, balance, and locomotion have been routinely noted in those in ASIA B‐D grades when rehabilitation strategies were focused on those outcomes (Cote et al., 2017), suggesting that there is a capacity to improve function after moderate SCI, likely leading to increased independent living and quality of life.

The preservation of neurological tracts across the spinal lesion are important for all physiological systems below the area of injury, with the musculoskeletal system being one of the most affected post‐SCI (Qin et al., 2010). Outside of its role in locomotion, skeletal muscle is the most abundant tissue in a normal healthy individual. It is the major storage site for amino acids and glycogen, and as a highly metabolic tissue, important for systemic glucose maintenance. Those with SCI face multiple secondary metabolic consequences (Bauman & Spungen, 2001) as chronic immobilization and disuse following more severe SCIs are coupled with reduced physical activity (Rocchi et al., 2017). The acute stage of SCI is problematic for skeletal muscle after both complete and incomplete injuries. The immediate disuse of skeletal muscle is accompanied by a proinflammatory environment from direct trauma and surgery, obligatory loss in nitrogen balance, and depending on severity, reduced central nervous system regulation (Thibault‐Halman et al., 2011). All these factors lead to rapid muscle atrophy (Castro et al., 1999; Talmadge et al., 2002) through a well‐conserved cellular program (Bodine, 2013).

One of the hallmarks of disuse atrophy is the oxidative‐to‐glycolytic fiber type transition in which slow contractile, fatigue‐resistant and mitochondria‐dense type I and IIa muscle fibers switch to fast contractile, fatigable and heavily glycolytic muscle type IIx (humans and rodents) and type IIb (rodents) fibers. The loss of mitochondria greatly impacts skeletal muscle. Healthy mitochondria buffer calcium, generate ATP and deliver substrates and metabolites through a vast reticulum of consistently fusing individual mitochondrion (Hood et al., 2016; Iqbal & Hood, 2015). Poorly functioning mitochondria generate detrimental reactive oxygen species (ROS), induce a mitochondrial‐specific autophagic program called mitophagy, and release factors which activate proteolytic signaling to induce muscle atrophy (Hyatt et al., 2019). Thus, targeting and protecting the function of mitochondria in the acute phase of a moderate severity SCI with a drug therapy may be a means to slow the rate of muscle atrophy.

SS‐31 [aka elamipretide (currently in Phase II and III clinical trials), formerly known as bendavia] is a promising tetrapeptide that strongly localizes to the mitochondria (Petri et al., 2006; Szeto & Birk, 2014). While originally thought to have antioxidant capacities, its primary mechanism of action has recently been demonstrated to be its interaction with surface charge densities and cardiolipin in the inner‐membrane of the mitochondria (IMM) where it enhances lipid packing and promotes bilayer curvature (Birk et al., 2013; Mitchell et al., 2020). Additionally, SS‐31 has been shown to directly interact with mitochondrial proteins associated with ATP production and improve respiratory function (Chavez et al., 2020). Of important clinical relevance, is it is well‐tolerated in humans (Eirin et al., 2019; Karaa et al., 2018). There is a strong pre‐clinical line of evidence demonstrating SS‐31 as efficacious to improving mitochondrial health and function in skeletal muscle in both pathological states and aging (Campbell et al., 2019; Min et al., 2011; Powers et al., 2011; Righi et al., 2013; Sakellariou et al., 2016; Siegel et al., 2013; Talbert et al., 2013). As specifically related to disuse, daily treatment with SS‐31 in mice has been able to prevent losses in soleus and plantaris muscle mass after 7 d (rats) (Talbert et al., 2013) and 14 d (mice) (Min et al., 2011) of hindlimb unloading, with associated markers of protected mitochondrial function. SS‐31 treatment can protect the function of the rat diaphragm in ventilator‐induced muscle weakness models (Powers et al., 2011) as well as prevent aging‐associated elevations in muscle fiber fluorescent ROS indicators and protein carbonylation markers (Sakellariou et al., 2016).

The role of limiting mitochondrial dysfunction during disuse atrophy is a logical therapeutic aim and SS‐31 has repeatedly been shown to have promising effects in protecting muscle health in disease models. To determine if SS‐31 could be a potential intervention to prevent muscle loss after SCI, we used a spinal cord contusion model to induce an incomplete SCI in young male mice and gave daily injections of SS‐31 or vehicle. We hypothesized animals treated with SS‐31 would have preserved muscle mass compared to vehicle‐treated animals 14 d post‐SCI.

## 2 MATERIALS AND METHODS

### 2.1 Animals

Male C57BL/6 mice aged 8 w were purchased from Charles River and allowed to acclimate for 1 week in a standard animal housing facility with a 12:12 light‐dark cycle before surgery. Male mice were used in this study as they comprise ~80% of all those with SCI ( National Spinal Cord Injury Statistical Center, 2020) and ~95% of the US Veteran SCI population (Curtin et al., 2012). Animals were split into three groups: a laminectomy‐only control group (Sham; n = 7), a laminectomy+contusion SCI treated with vehicle‐only (SCI+Veh; n = 8) and a laminectomy+contusion SCI treated with the drug SS‐31 (SCI+SS‐31; n = 8). The vehicle for this study was lactated Ringer's solution. All animals were provided ad libitum access to standard chow and water and were given daily fruit crunch treats during the course of the study. Food and treats were kept on the cage floor throughout the study to ensure easy access for paralyzed animals. All studies were reviewed and approved by the Institutional Animals Care and Use Committee at the University of Alabama‐Birmingham (IACUC #: 21639).

### 2.2 Laminectomy and contusion SCI

All animals were weighed before surgery and then anesthetized using 2%–5% isoflurane. The area over the spine was shaved with clippers, cleaned with 70% ethanol, and then covered with a betadine solution. A midline incision was made over the areas of T7‐T11 and the muscle along the spinal column was removed from T7 to T11 to expose the vertebrae and to allow a clean area for vertebral clamping. T9 was removed by carefully cutting each side of the dorsal process with sharp surgical scissors and the vertebral arch was removed in one piece using fine forceps. The sham animals promptly had the muscles along the spinal column sutured together over the laminectomy site to provide support and muscle tension for the spine then the incision site was closed with surgical wound clips. Animals selected for a contusion SCI were clamped into position using locking forceps provided with the Infinite Horizon Impactor. A contusion SCI model was used as it would be expected the paralysis and reduced locomotor function after SCI, coupled with the systemic stress of the injury itself, would lead to muscle atrophy and mitochondrial dysfunction. Once the animal was properly placed and aligned, an Infinite Horizon Impactor probe was positioned over the exposed spinal cord and a 50 kdyne injury force with 0 s dwell was applied. The animals were quickly removed from the clamps and equal bilateral bruising of the spinal cord was visually confirmed before suturing and wound clipping. All animals were placed in clean cages filled with AlphaDri+ bedding on warming pads and were singly‐housed for the rest of the study.

### 2.3 Post‐operative care and tissue harvest

Animals were administered a 1.0 ml cocktail containing 5.0 mg/kg of carprofen and 0.1 mg/kg buprenorphine in lactated Ringer's solution immediately after surgery, with the SS‐31 animals receiving 5.0 mg/kg SS‐31 in their cocktail. SS‐31 was commercially made by Genscript and the dosage of SS‐31 used for this study was selected because in pilot studies using a sciatic nerve transection model, we noted no difference in hindlimb muscle mass in animals given daily injections of 1.5 mg/kg SS‐31 or vehicle for 14 d (S1 Fig: https://www.doi.org/10.6084/m9.figshare.13232564). 5.0 mg/kg was further selected to match the highest published concentration used for studies focusing on muscle (Lee et al., 2011) although no toxicity has been noted in mice with doses up to 50 mg/kg (Petri et al., 2006). An additional administration of 5.0 mg/kg of carprofen was given 24 h post‐surgery and additional 0.1 mg/kg buprenorphine injections were administered to all animals every 12 h for 3 d. SS‐31 and vehicle were administered daily and bladders were manually expressed 2–3 times a day.

At the time of sacrifice, mice were anesthetized with 2–5% isoflurane and the area around both hindlegs was shaved and cleaned with 70% ethanol. The soleus, plantaris, gastrocnemius, tibialis anterior (TA), and extensor digitorum longus (EDL) were carefully removed and wet weights were determined. The left soleus and plantaris were prepared for ex vivo protein synthesis studies and the left TA was mounted in an OCT/tragacanth gum mixture and flash‐frozen in liquid nitrogen‐cooled isopentane for immunohistochemical analysis. All other muscles were flash frozen in dry‐ice cooled isopentane. Animals were euthanized by the combination of exsanguination and removal of the heart.

### 2.4 Ex vivo protein synthesis

Protein synthesis was measured using the surface sensing of translation method (SUnSET) (Goodman et al., 2011) with minor modifications. Briefly, the left soleus and plantaris were carefully extracted then placed in lactated Ringer's solution on wet ice for 10 min. The muscles were then removed and placed in a solution of Krebs‐Henseleit buffer supplemented with 1x modified essential amino acids and 25 mM glucose and incubated for 20 min at 37°C. The samples were then rinsed in warmed PBS and placed in a solution of serum‐free DMEM containing 10 µM puromycin for 30 min at 37°C. Muscles were blot‐dried on gauze and then flash‐frozen in liquid nitrogen‐cooled isopentane for storage.

### 2.5 Tissue homogenization and protein immunoblotting

The soleus and plantaris muscles used from the SUnSET studies were homogenized in 1x RIPA buffer supplemented with a protease and phosphatase inhibitor cocktail (Halt 100x Protease and Phosphatase Inhibitor) using a bead‐mill homogenizer. The homogenates were put on wet ice for 30 min and then spun at 14,000 *g* for 20 min at 37°C. The supernatant was collected and protein concentrations were detected using a microBCA kit (Pierce). Electrophoresis and immunoblotting were completed using the Wes automated 12–230 kDa capillary electrophoresis system as recommended by the manufacturer (ProteinSimple).1.2 µg of protein was loaded from each sample and then separated for 25 min at 375 V. Blocking lasted 30 min, followed by a 60 min incubation with an anti‐puromycin antibody (1:100 dilution; Clone 12D10, Millipore Sigma) for 60 min. The secondary antibody timeframe was 30 min and was used as directed by the manufacturer (Mouse Kit, ProteinSimple). After chemiluminescence detection, whole lane densitometry values were determined from the 12–230 kDa range of the High Dynamic Range Image using the Drop Down algorithm available in the Compass software program (ProteinSimple). All samples were normalized to whole‐lane total protein values determined using methods identical as above using a total protein detection kit (ProteinSimple). Original Compass files, as well as unaltered and uncropped images of all immunoblots can be found at https://www.doi.org/10.6084/m9.figshare.12719099.

### 2.6 Protein carbonyl determination

Protein oxidation as determined by protein carbonyl levels was measured using 20–25 mg of gastrocnemius tissue homogenized similar to the above methods, with the gastrocnemius being cut transversely along the belly to capture equal portions of both the medial and lateral portions. 20 µg/ml of muscle homogenate was then derivatized using dinitrophenylhydrazine and protein carbonyl concentrations were determined by ELISA (OxySelect Protein Carbonyl ELISA Kit; Cell BioLabs, cat #: STA‐310) following the manufacturer's instructions.

### 2.7 RNA isolation and RT‐qPCR

20–25 mg of the left gastrocnemius was cut from equal portions of the medial and lateral sections then homogenized in 700 µl of Qiazol using a bead‐mill homogenizer and then phase separated using chloroform and centrifugation at 14,000 *g* for 15 min at 4°C. The clear phase was removed and mixed with 100% ethanol and RNA was isolated following the manufacturer's guidelines (miRNeasy mini‐kit, Qiagen). RNA concentrations and 260:280 ratios were determined using the Nanodrop system and 1 µg of RNA was used to create a cDNA library (High Capacity RNA‐to‐cDNA kit, Applied Biosystems). The cDNA library was diluted 1:10 with nuclease‐free water and gene expression was determined using Taqman gene expression assays following the manufacturer's guidelines (Applied Biosystems). The complete list and catalog numbers of gene expression assays can be found S1 Table (https://doi.org/10.6084/m9.figshare.12400898). Comparisons of gene expression were completed using the 2^−ddCt^ method with beta‐2‐microglobin (B2M) used as the housekeeping gene. The average Ct for   B2M for each group was: Sham=21.920, SCI+Veh = 21.913, SCI+SS‐31 = 21.777.

### 2.8 Immunohistochemistry

Muscle fiber cross‐sectional area (fCSA) was determined via unblinded manual tracings of 150 fibers/sample by an investigator (ZAG) after immunohistochemistry using laminin staining of the TA muscle. 10 µm sections were cut using a cryostat, dried for 30 min at room temperature and then stored at −20°C. To prepare for immunohistochemistry, frozen samples were thawed for 10 min at 37°C in a humidity chamber and rehydrated at room temperature with 2 × 5 min incubations in PBS. Slides were fixed for 3 min in a 1:1 solution of acetone and methanol, rinsed quickly in PBS, then placed in 2 × 5 min PBS washes. Sections were then blocked for 20 min in a 5% goat serum and PBS solution, washed for 5 min in PBS, and placed in a 1:400 antibody solution of rat anti‐laminin a/b (MA1‐06100, Thermo) in a 1% goat serum and PBS solution for 30 min at 37°C. The antibody mixture was aspirated off and quickly rinsed twice in PBS then washed 3 × 5 min in PBS. The cross‐sections were then incubated with a secondary antibody solution containing an anti‐mouse Alexa488 antibody (A‐21121, Thermo) in 1% goat serum in PBS at 37°C for 30 min. Cross‐sections were again quickly rinsed twice in PBS then washed 3 × 5 min in PBS. They were then incubated for 2 min at room temperature in a 1:200,000 solution of Hoescht stain in PBS to counter‐stain the nucleus, rinsed twice in PBS, washed 3 × 5 min in PBS, then allowed to dry for 5 min. Mounting media was placed on the samples and a coverslip was sealed in place using standard fingernail polish. Images were captured using stitched images at 20x using an Olympus BX51 fluorescent microscope with MagnaFire SP Camera. Image analysis was completed using Image‐Pro Plus 9.3 software. One sample from the SCI+SS‐31 group was omitted due to the poor quality of the mounted muscle.

### 2.9 Cell culture and oxygen consumption rates

Cell culture assays were used to determine the efficacy of the commercially made SS‐31. C2C12 cells were seeded at a 20,000 cell/well density in a Seahorse XF cell culture microplate and allowed to adhere for 24 h in standard DMEM with 10% fetal bovine serum (FBS) and 1% penicillin/streptomycin. Cells were then treated with 10 µM of morpholino targeting calstabin1 or a control morpholino (Gene Tools, LLC) for 48 h. Reductions in calstabin1 create a ‘leaky’ sarcoplasmic reticulum by preventing its proper binding and gating of the ryanodine receptor 1 (RyR1) (Andersson et al., 2011). This increases the open probability of the RyR1 and allows for calcium leak and cytosolic calcium accumulation, which can decrease muscle and mitochondrial health (Andersson et al., 2011; Bellinger et al., 2009) and, in cardiac tissue, lead to mitochondrial dysfunction through sustained mitochondrial calcium overload (Santulli et al., 2015). Cells were washed three times in 1x PBS and then pre‐incubated at 0% CO_2_ and 37°C for 1 h with 5 µM SS‐31 or vehicle (PBS) in Seahorse XF DMEM (Agilent) with 1 mM pyruvate, 2 mM glutamine and 10 mM glucose. Cells were then loaded into a Seahorse XFe96 and oxygen consumption rates (OCR) were tested using the Seahorse XF Cell Mito Stress Test kit (Agilent) with oligomycin (final concentration: 0.5 µM), FCCP (final concentration: 2.0 µM) and rotenone/antimycin A (final concentration: 0.5 µM). Baseline respiration (basal OCR – rotenone/antimycin A OCR), coupled respiration (basal OCR – oligomycin OCR), maximal respiration (FCCP OCR – rotenone/antimycin A OCR), and oxidative reserve (FCCP OCR – basal OCR) were the measured outcomes. Two independent experiments were run with 9 wells/group with the wells with the highest and lowest average oxygen consumption being excluded *a priori* from each group to limit variability due to excessive or poor automated well injection. Thus, a total of 7 wells/group per experiment were used (14 wells/group). All values were normalized to number of DAPI‐stained nuclei captured by an EVOS FL Auto Imaging System (Invitrogen) and counted using Celleste Image Analysis software (Invitrogen).

### 2.10 Statistics

Body weight measures were compared using a two‐way mixed model ANOVA with Tukey's multiple comparisons. We chose to focus *a priori* on group differences at each time point as well as their respective pre‐surgery weight. Cell culture outcomes and muscle fCSA distribution bins (500 µm bins) were also analyzed with a two‐way mixed model ANOVA with the focus of fCSA being on differences between groups per bin. Other variables were compared using one‐way ANOVAs with Tukey's multiple comparisons follow‐up tests as necessary. Data are presented as means +/‐ 95% confidence interval with effect size represented with *eta^2^* values and corresponding ANOVA *p* value. Column means are shown in bold at the bottom of each bar chart. A threshold to determine statistical differences among groups was set at *p* < 0.10, though we acknowledge there are limitations to statistical analyses based on null hypothesis testing (McShane et al., 2019). Meaningful group differences are noted with brackets between groups and the *p* value from Tukey's follow‐up test. For clarity, only meaningful differences are presented for the gene expression assays. All statistical tests were calculated using Prism 8.0 (GraphPad) and every statistical output from the study can be found in S2 Table (https://www.doi.org/10.6084/m9.figshare.12400904). All data sets used to generate the figures and tables for this manuscript can be found at https://www.doi.org/10.6084/m9.figshare.12719678.

## 3 RESULTS

### 3.1 C2C12 oxygen consumption rates

There was a Drug x Morpholino interaction for baseline OCR (S2A Fig.; interaction effect: *p* = 0.034, main effect of Drug: *p* = 0.023, main effect of Morpholino: *p* = 0.800), coupled OCR (S2B Fig.; interaction effect: *p* = 0.020, main effect of Drug: *p* = 0.051, main effect of Morpholino: *p* = 0.793), maximal OCR (S2C Fig.; interaction effect: *p* = 0.012, main effect of Drug: *p* = 0.006, main effect of Morpholino: *p* = 0.586), and oxidative reserve (S2D Fig.; interaction effect: *p* = 0.033, main effect of Drug: *p* = 0.018, main effect of Morpholino: *p* = 0.579). Simple effects comparisons showed elevations in all OCR outcomes in the SS‐31‐treated cells with the calstabin1 knockdown compared to vehicle‐treated cells with the calstabin1 knockdown. Additionally, maximal OCR was elevated in the SS‐31‐treated calstabin1 knockdown cells compared to the control morpholino cells treated with vehicle. S2 Fig can be found at https://www.doi.org/10.6084/m9.figshare.13522766.

### 3.2 Body mass

**FIGURE 1:**
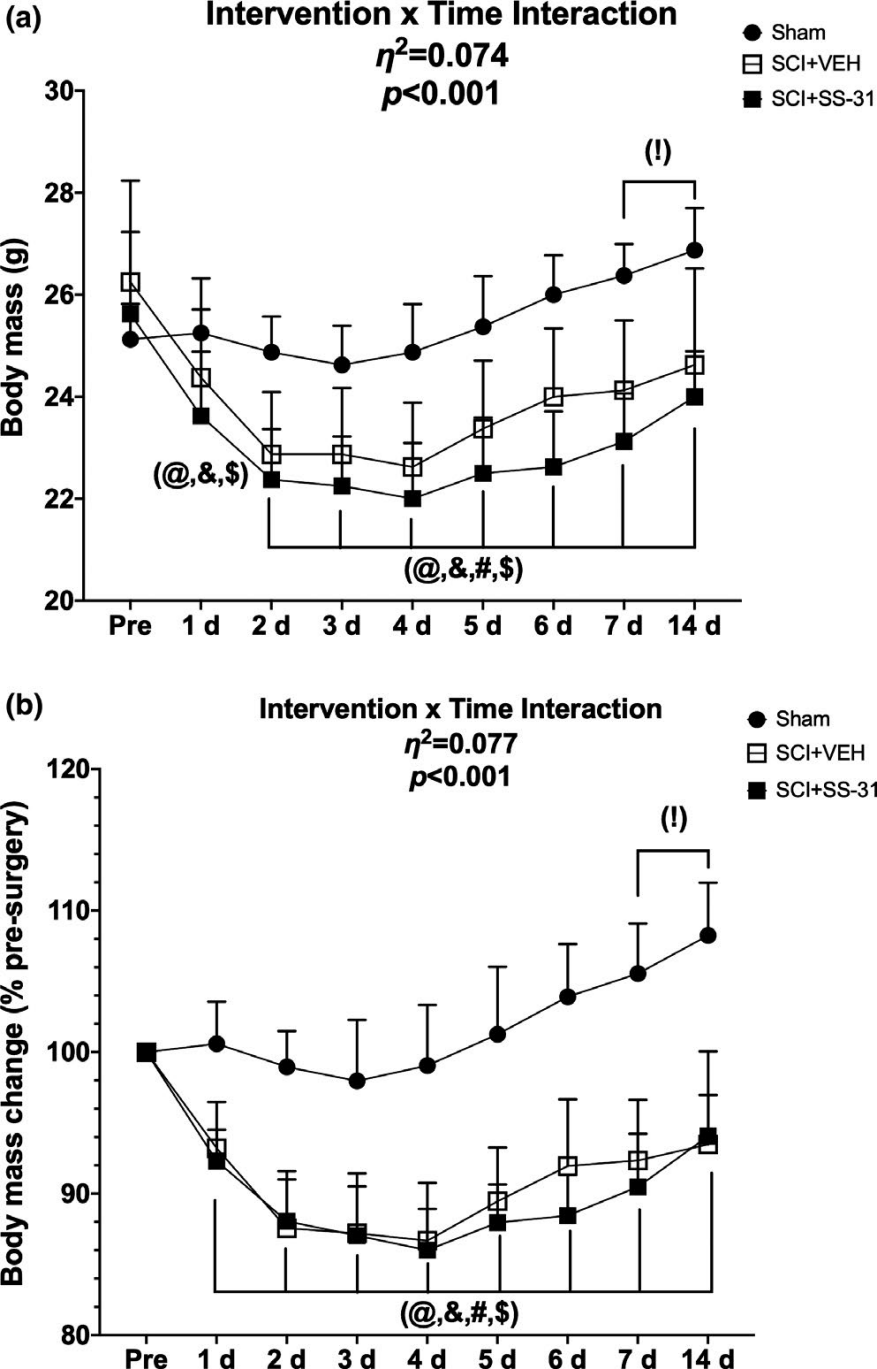
(a) Absolute body mass and (b) relative body mass across 14 d after a sham or contusion SCI with or without SS‐31. Data are presented as means with the upper bound of a 95% confidence interval. (!) denotes a within group difference for Sham compared to its Pre‐ timepoint, while (@) and (&) represent similar within group differences for SCI+Veh and SCI+SS‐31, respectively, compared to their Pre‐ timepoint. (#) denotes between group differences between Sham and SCI+Veh and ($) denotes between group differences between Sham and SCI+SS‐31. Exact *p* values for simple effects can be found in S2 Table.

There was Surgery x Time interaction for both absolute body mass (Figure 1a; interaction effect: *p* < 0.001, main effect of Surgery: *p* = 0.010, main effect of Time: *p* = 0.002) and relative percent change (Figure 1b; interaction effect: *p* < 0.001, main effect of Surgery: *p* < 0.001, main effect of Time: *p* < 0.001). Multiple comparison testing of the simple main effects showed the SCI‐SS‐31 group as having reduced absolute body mass compared to Sham starting at 1 d post‐surgery, with the SCI‐Veh group being reduced starting at 2 d post‐surgery, with no effect of SS‐31 between SCI groups. Both SCI groups had relative body mass losses starting at 1 d post‐surgery, with no effect of SS‐31. These group differences were sustained at each measurement until the end of the study. Similarly, when doing follow‐up testing to determine within‐group differences of the interventions compared to each group's respective Pre‐ timepoint, the SCI groups showed losses starting at 1 d post‐SCI, while the Sham animals were able to gain absolute and relative mass by 7 d post‐intervention. Again, there was no effect of SS‐31 for any of these comparisons. The exact *p* values for each simple main effect comparison can be found in S2 Table.

### 3.3 Tissue mass

**FIGURE 2:**
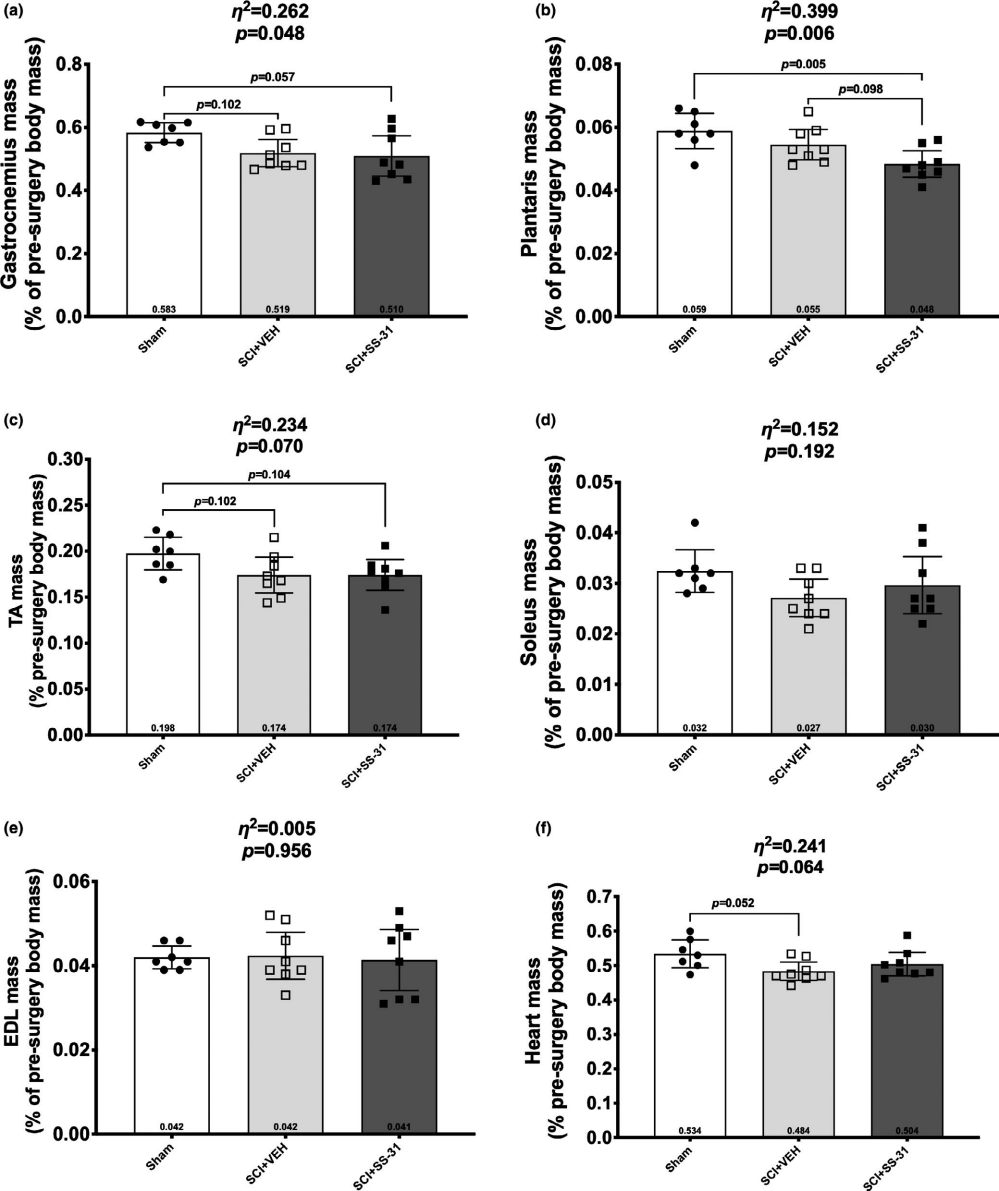
Bar graphs showing individual group differences for (a) gastrocnemius, (b) plantaris, (c) tibialis anterior, (d) soleus, (e) extensor digitorum longus and (f) heart muscles. Data are shown as mean +/‐ 95% confidence interval with notable mean differences between groups indicated by brackets and Tukey's multiple comparison *p* values.

There was a reduction in the mass of the gastrocnemius (Figure 2a; *p* = 0.048) with multiple comparisons tests showing the SCI+SS‐31 animals having lower muscle weights than Sham animals. There was also a loss in plantaris mass (Figure 2b; *p* = 0.006) with the SCI+SS‐31 group having reduced mass compared to the Sham and a SCI+Veh groups. There was a difference between groups in TA mass (Figure 2c; *p* = 0.070), though follow‐up multiple comparisons between groups had *p* values above our statistical threshold. There were no changes seen in the soleus (Figure 2d; *p* = 0.192) or EDL (Figure 2e; *p* = 0.956). Lastly, there were differences in heart mass (Figure 2f; *p* = 0.064), with the SCI+Veh group being reduced compare to sham animals.

### 3.4 Tibialis anterior fCSA

**FIGURE 3:**
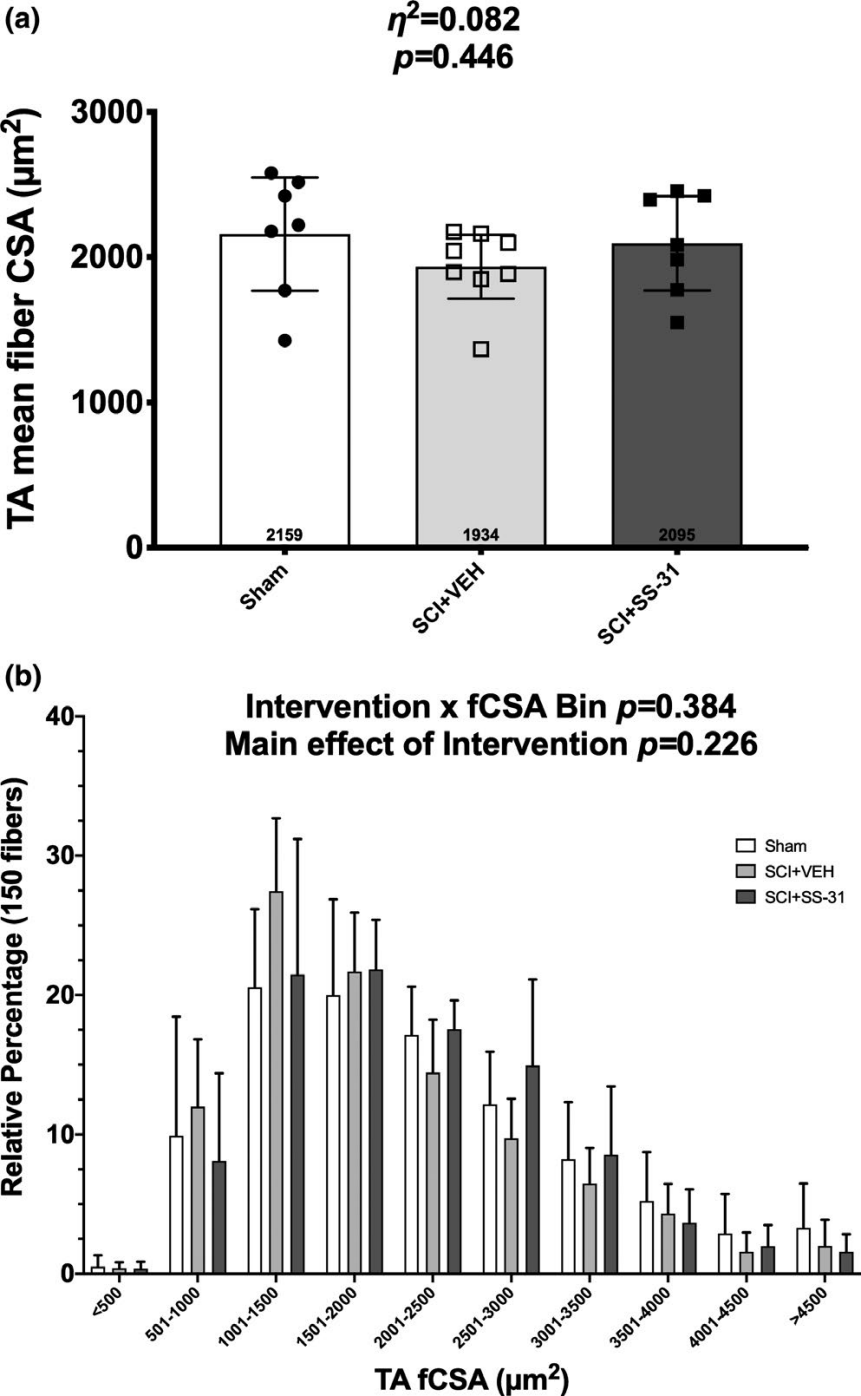
Muscle fiber cross‐sectional area (fCSA) of 150 fibers/sample from the tibialis anterior muscle represented by (a) mean overall fCSA and (b) 500 µm^2^ bin fiber size percentage 14 d after a laminectomy or contusion SCI with or without a daily SS‐31 treatment. Data are shown as means +/‐ 95% confidence interval for the overall mean and the upper limit of the 95% confidence interval for the distribution panel to help with clarity of the data.

There was no difference in average fCSA of the TA between groups (Figure 3a; *p* = 0.446) and there were no interaction or relevant main effects for the relative percentage of fiber size between groups (Figure 3b; *p* = 0.226).

### 3.5 Protein carbonylation and protein synthesis

**FIGURE 4:**
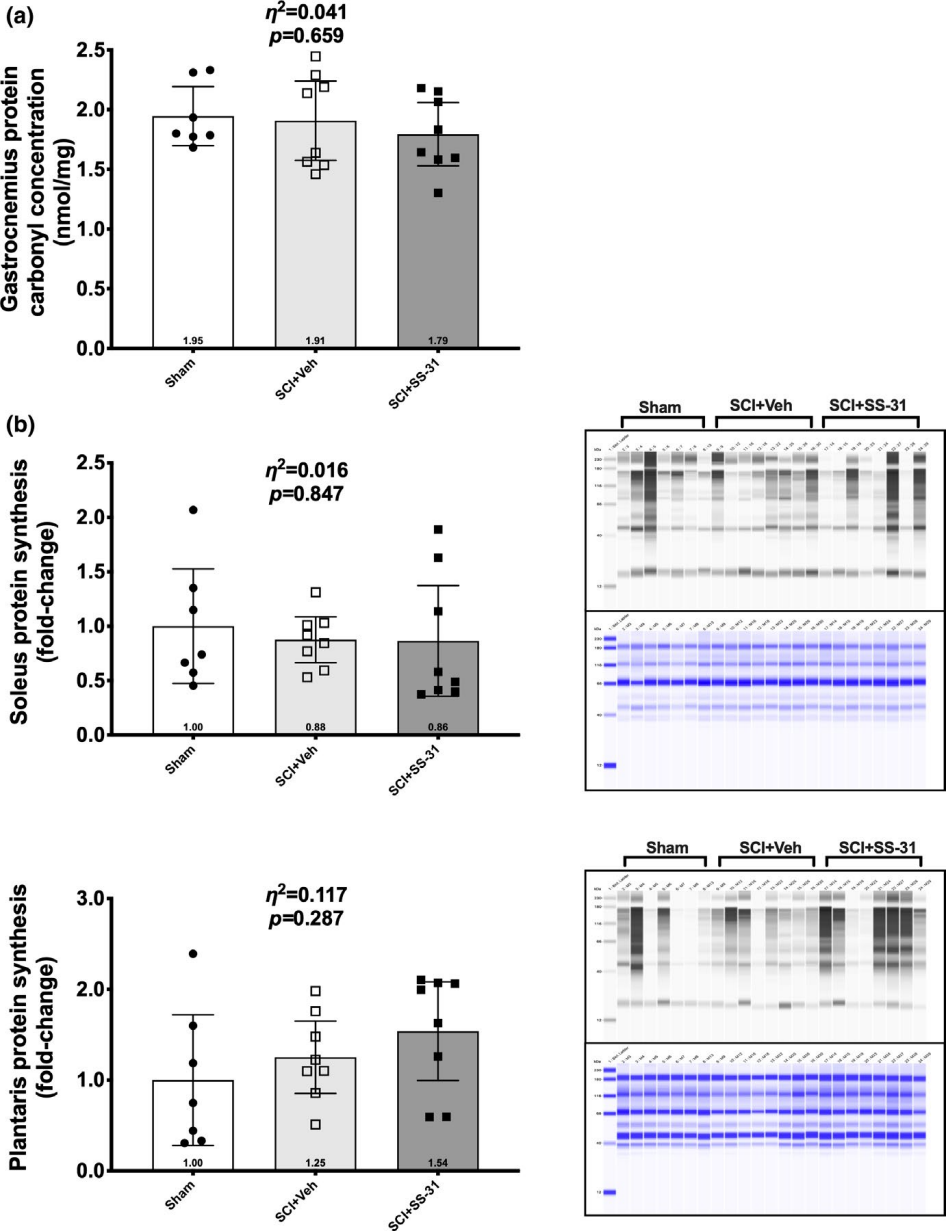
Protein carbonyl and synthesis measurements using ELISA or the *ex vivo* SUnSET method respectively. (a) Protein carbonyl levels of whole muscle homogenate from the gastrocnemius. Protein synthesis markers of the (b) soleus and (c) plantaris muscle groups in Sham, SCI+Veh or SCI+SS‐31 animals 14 d after surgery. Data are shown as the mean +/‐ 95% confidence interval with all samples present in both the immunoblot image (black and white) and total protein image (blue).

There were no differences in concentrations of protein carbonylation between groups (Figure 4a; *p* = 0.659). Additionally, there were no group differences in protein synthesis in the soleus (Figure 4b; *p* = 0.847) or plantaris (Figure 4c; *p* = 0.287).

### 3.6 Gene expression

**FIGURE 5:**
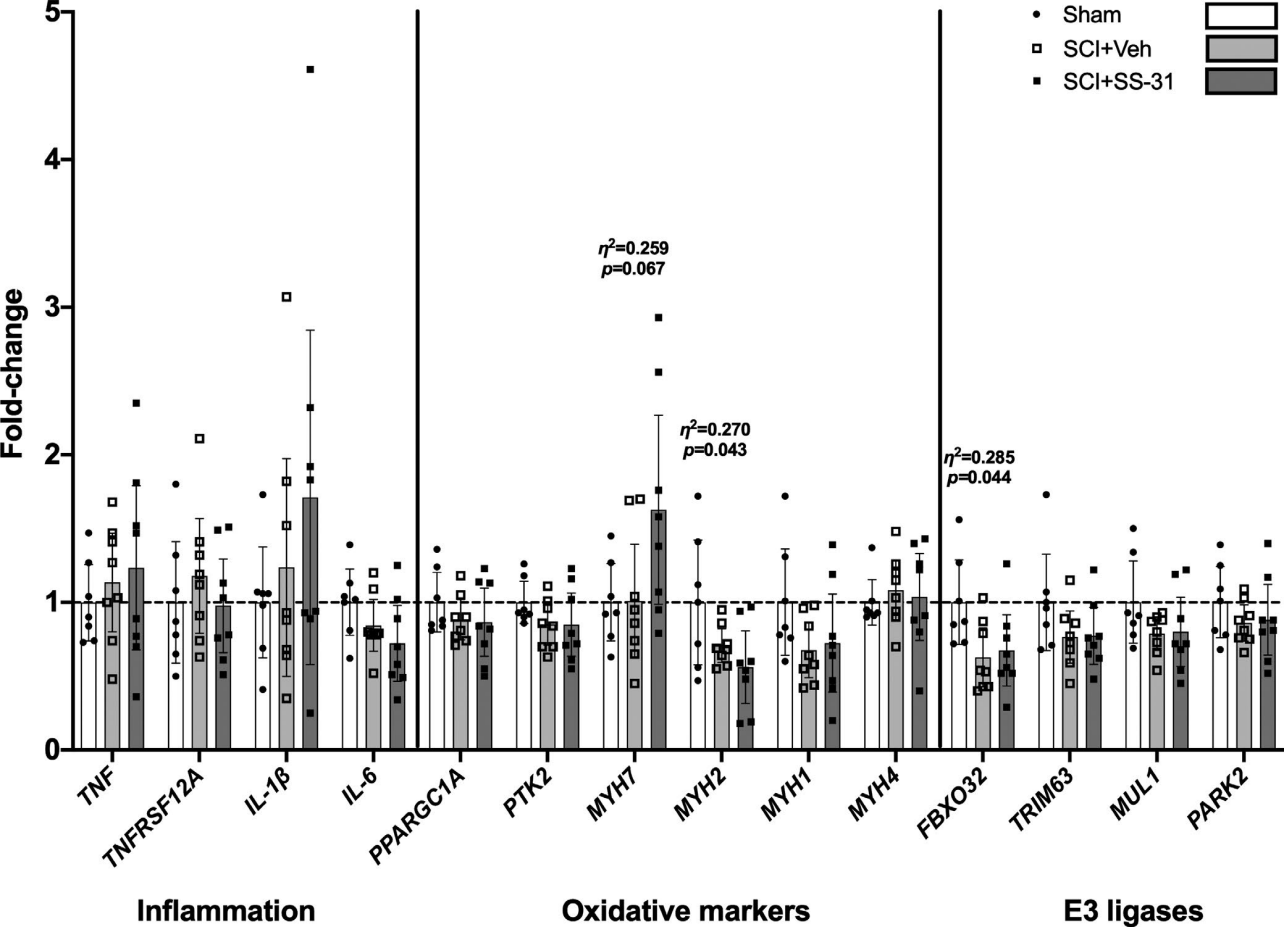
Gastrocnemius mRNA expression of well‐known genes associated with inflammation, oxidative state of the muscle, and E3 ligases. Data are shown as the mean +/‐ 95% confidence interval.

All gene expression assays are presented in Figure 5 with statistical comparisons shown in Table 1. There were no differences in gastrocnemius gene expression of the pro‐inflammatory associated *TNF* (*p* = 0.649) or *TNFRSF12A* (Fn14; *p* = 0.604), *IL1B* (*p* = 0.374) or *IL6* (*p* = 0.137), among groups. Additionally, there was no difference in *PPARG1A* (*p* = 0.470) or *PTK2* mRNA expression (*p* = 0.277). mRNA for the type I myosin heavy chain *MYH7* was altered between groups (*p* = 0.067) with the SCI+SS‐31 group being elevated compared to sham animals. There were also group differences in mRNA expression of the type IIa myosin *MYH2* (*p* = 0.043) with SCI+SS‐31 being reduced compared to Sham. There were no differences noted in mRNA expression of *MHY1* (*p* = 0.174) or *MYH4* (*p* = 0.839). There was a reduction in the mRNA expression in SCI animals for the E3 ligase *FBXO32* [MAFbx (*p* = 0.044)] with no major differences in expression of *TRIM63* [MuRF1 (*p* = 0.189)], *MUL1* (*p* = 0.208) or *PARK2* (*p* = 0.491).

**TABLE 1:** Main statistical outcomes and follow‐up multiple comparisons testing for gene expression assays carried out in Figure 5.

Gene	*Eta^2*	ANOVA *p* value	Tukey's between groups adjusted *p* value
*TNF*	0.042	0.649	n/a
*TNFRSF12A*	0.049	0.604	n/a
*IL‐1β*	0.094	0.374	n/a
*IL‐6*	0.181	0.137	n/a
*PPARGC1A*	0.073	0.470	n/a
*PTK2*	0.121	0.277	n/a
*MYH7*	0.249	0.057	Sham vs. SCI+SS−31 (*p* = 0.097); SCI+Veh vs. SCI+SS−31 (*p* = 0.089)
*MYH2*	0.270	0.043	Sham vs. SCI+SS−31 (*p* = 0.036)
*MYH1*	0.161	0.174	n/a
*MYH4*	0.017	0.839	n/a
*FBOX32*	0.280	0.037	Sham vs. SCI+Veh (p = 0.044); Sham vs. SCI+SS−31 (*p* = 0.085)
*TRIM63*	0.153	0.189	n/a
*MUL1*	0.145	0.208	n/a
*PARK2*	0.069	0.491	n/a

## 4 DISCUSSION

We used a moderate 50 kdyne thoracic contusion SCI on 9‐week old male mice and monitored them out to 14 d post‐injury to determine if the mitochondrial‐targeting peptide SS‐31 could prevent the loss of muscle and body mass typically seen in mice post‐SCI. This model of SCI resulted in decreased body mass and the loss in mass of plantaris and gastrocnemius muscles, while the administration of SS‐31 had no protective effect on these outcomes. There was no effect of SCI with or without SS‐31 on markers of protein synthesis or protein carbonylation, or on inflammatory cytokine gene expression. However, SS‐31 was associated with alterations in the mRNA expression profile of the slow twitch type I and fast‐oxidative type IIa myosin heavy chain. Taken together, SS‐31 does not seem to be an efficacious therapy to prevent body or muscle mass losses after a 50 kdyne contusion SCI in mice.

Muscle atrophy occurs rapidly in female mice after a complete SCI as 20–25% of gastrocnemius mass is lost by 7 d with additional losses seen at 28 d post‐SCI (Graham et al., 2019). A complete transection rarely occurs in humans, however, and incomplete SCI is likely to be associated with varying amounts of atrophy depending on injury severity and recovery and rehabilitation. Those with incomplete SCI may have U‐shaped curves for muscle mass and function over time as unloaded muscle undergoes catabolism during the acute time frame post‐injury followed by recovery of muscle mass and function due to spontaneous neurological recovery and sustained reloading (National Spinal Cord Injury Statistical Center, 2020). Our data indicate that a 50 kdyne contusion injury of 9‐week old male mice is likely not a suitable model for inducing sustained muscle atrophy. A caveat to the interpretation of our data is that muscles were studied on the ‘upswing’ of the U‐curve and at a time where neurorecovery has occurred. However, our data suggests that this model is still viable for inducing SCI‐associated systemic stress as noted by the consistent loss in body mass in the SCI groups.

Body mass loss in the acute phase of SCI is a common outcome in both complete and incomplete pre‐clinical (Graham et al., 2016; Otzel et al., 2019; Scholpa et al., 2019) and clinical injuries (Powell et al., 2017; Thibault‐Halman et al., 2011). In pre‐clinical models, this is due to muscle atrophy and the loss of body fat, which has been reported after a complete spinal cord transection (Graham et al., 2016; Primeaux et al., 2007) and after a light‐moderate contusion SCI in rats (Harris et al., 2019), though this loss is not as severe. While food intake was not monitored in this study, the animals had continual access to food and treats placed on the cage bottom. Food consumption of chronically transected SCI animals is the same or slightly greater than sham controls (Primeaux et al., 2007) but cumulative caloric intake is roughly 10–15% lower in rats with a contusion SCI in the acute weeks after injury (Harris et al., 2019). Thus, we cannot rule out that reduced food consumption was a primary driver of the body mass loss. Additional likely factors for this loss are stress from the trauma itself alongside any related surgical procedure, reduced drinking despite ad libitum access even while paralyzed, disruption in thermoregulation as well as muscle disuse and possible hypermetabolism (Harris et al., 2019; Thibault‐Halman et al., 2011). Our model of moderate SCI is consistent with the pre‐clinical literature as we show sustained body mass loss compared to sham controls across 14 d (Bigford et al., 2018; Graham et al., 2020).

SS‐31, a small tetrapeptide that targets cardiolipin in the IMM of the mitochondria, has consistently been demonstrated to have beneficial skeletal muscle outcomes in various models of muscle wasting associated with mitochondrial dysfunction. In aged mice, SS‐31 increased levels of oxidative phosphorylation coupling, muscular endurance of the TA muscle, and treadmill running capacity (Siegel et al., 2013). In another aging model, SS‐31 was associated with improvements in mitochondrial health as noted by reduced H_2_O_2_ release, fluorescence ROS indicators, and post‐translational markers of oxidative stress in aged muscle (Sakellariou et al., 2016). More relevant to our studies is disuse atrophy. In mouse hindlimb muscle denervated from a sciatic nerve transection, mitochondrial‐derived ROS are substantially elevated by 7 d post‐paralysis, demonstrating rapid mitochondrial dysfunction (Bhattacharya et al., 2009; Muller et al., 2007). In a mouse model of bilateral cast immobilization, daily treatment with 1.5 mg/kg of SS‐31 prevented losses in soleus and plantaris mass. Additionally, SS‐31 was able to prevent the generation of ROS in the soleus and limit its generation in the plantaris (Min et al., 2011). Our results diverge from the literature as our SCI model did not result in any appreciable loss in mass of the soleus or EDL muscle or substantially reduce the fCSA of the TA. The commercially prepared SS‐31 used in this study was able to improve oxygen consumption in C2C12 cell culture assays with a morpholino‐induced knockdown of calstabin1, showing the drug was bioactive and an important finding considering we noticed no clear beneficial effect of SS‐31. In the muscles for which we noted consistent whole muscle losses in the SCI groups, such as the gastrocnemius, and plantaris, there was no effect of SS‐31. Reduced mRNA levels of *Fbox32* (MAFbx) and the lack of differences in the other E3 ligases MuRF1, Mul1, and Parkin in our animals suggests that they are in a period of consistent reloading or have returned to normal as typical disuse leads to a quick upregulation of these ligases and a return to baseline by 14 d in rodents (as reviewed in Bodine & Baehr (2014). The discrepancy in the muscle weight data of this report compared to the literature is likely due to differences in the disuse model. Cast immobilization preserves the integrity of communication between the immobilized muscle and CNS while our model partially disrupts it by creating a spinal cord lesion followed by some recovery. Our protein carbonyl levels suggest that SCI, with or without SS‐31, was not associated with excessive oxidative stress as levels between groups were similar to those of Sham animals. The neurorecovery and return of locomotor function and muscular loading following a contusion SCI of this severity may be overshadowing any SS‐31‐induced protection in muscles below the area of spinal injury. It is also possible that SS‐31 had no effect on our muscles as they were largely back to a baseline level of function and SS‐31 has minimal effects on healthy mouse skeletal muscle (Min et al., 2011).

While we did not quantify locomotor recovery, all mice that received a contusion SCI were weight‐bearing between 4–7 d after injury and all were walking by 10–14 d, consistent with other studies using a 50 kdyne contusion SCI (Bottai et al., 2008). The timeline of recovery for neurological and locomotor outcomes after a moderate contusion SCI likely has an important role in regulating the physiological state and health of paralyzed muscles. The moderate contusion force leaves more spared tracts across the injury site and maintains the innervation of muscle, limiting muscle atrophy. The young age of our mice (9 weeks old at surgery, 11 weeks old at sacrifice) may have meant that they were still in their growth phase, with continued growth providing some potential protection against muscle atrophy. Perhaps more importantly, the biochemical window of functional paralysis may be limited as animals begin to consistently load the hindlimb muscles by one week after injury as neuroplasticity processes begin. These discrepancies in the timeline of recovery between the SCI animals in our study likely had a major role in the wide range of responses in protein synthesis, as the mice would have had varied days of reloading. The major variation of protein synthesis in the sham animals has no clear explanation but access to food and fruit treats may have led to animals eating soon before sacrifice. We are not aware of any study that has looked into direct markers of protein synthesis in muscle following SCI but there seem to be discrepancies in this outcome depending on the muscle and model of disuse. Reports using the SUnSET method of detecting protein synthesis show increased gastrocnemius protein synthesis (You et al., 2015) with no change in TA protein synthesis (You et al., 2018) at 2 d post‐surgery in denervation models. However, protein synthesis was elevated in denervated TA muscles at 14 d post‐surgery (Watt et al., 2015). Splint‐induced immobilization has been shown to be sufficient to greatly reduce protein synthesis in adult female soleus and plantaris mouse muscles as early as 3 d with sustained reductions in protein synthesis levels out to 7 d (Quy et al., 2013). There should be some caution in trying to extrapolate differences in protein synthesis outcomes among muscle groups. Comparing data from the soleus or plantaris against the TA, which as a dorsiflexor has a different loading profile compared to the gastrocnemius which is the major load bearing muscle of the hindlimb, may provide confounding differences that are not representative of the biochemical state or function of the muscle. Additionally, fiber type differences between the muscle groups likely affect how these muscles respond to paralysis and reloading. Studies focusing on protein synthesis in muscle following SCI would likely have more consistent outcomes at moderate injuries in the immediate days (3–5 d) post‐injury when muscles below the lesion are still relatively unloaded or following a more severe injury force, which allows for a longer period of muscle disuse.

We have previously reported elevations in the mRNA of proinflammatory cytokines in the gastrocnemius at 56 d post‐complete SCI in female mice (Graham et al., 2016) and the first 1–2 m, but not 14 d, post‐severe contusion SCI in the soleus of male rats (Graham et al., 2020). Additionally, SS‐31 has been shown to prevent TNFα‐induced release of cytokines from 7 d differentiating myotubes (Lightfoot et al., 2015) suggesting that it may have a role in altering these factors in paralyzed muscle. Our data supports the latter study in that SCI, as well as SS‐31, was not associated with any changes in mRNA of pro‐atrophy cytokines in the early weeks post‐SCI, with the limitation being the mice in this study regained the ability to consistently load after the injury. A more extended period of muscle disuse or increase in the injury severity may be able to promote gene expression of pro‐inflammatory cytokines with a therapeutic window for SS‐31 as 7 d (Cea et al., 2013) and 14 d (Choi et al., 2018) of denervation were able to increase *IL1B*, *IL6*, and *Tnf* mRNA expression in gastrocnemius whole muscle homogenate.

Many models of disuse atrophy have been thoroughly investigated and the molecular changes and pathways were extensively described (Bodine, 2013; Ruegg & Glass, 2011) with one of these key outcomes being the oxidative‐to‐glycolytic fiber type shift (Ciciliot et al., 2013; Schiaffino et al., 2013; Schiaffino & Reggiani, 2011). There was no clear transition on mRNA expression from oxidative‐to‐glycolytic fiber types in the SCI+Veh animals. However, we do show that the SS‐31 treatment was associated with a reduction in mRNA for the MHC of the fast‐oxidative type IIa fiber (*MYH2*) and an upregulation of the mRNA for the MHC of the slow oxidative type 1 fiber (*MYH7*). How SS‐31 may be regulating this is unknown as there was not an associated change with PGC1α mRNA expression. 14 d of a 1.5 mg/kg SS‐31 treatment was not associated with clear changes in muscle fiber type size or permeabilized muscle oxidative respiration function in the soleus or plantaris of healthy mice compared to saline‐treated controls (Min et al., 2011), suggesting that SS‐31 has little effect on healthy muscle. However, our SS‐31 treatment dose was 5.0 mg/kg and this higher concentration may promote some response in muscle. One possible explanation is the consistent presence of SS‐31 binding to the IMM that may be change the molecular functioning in the cell during disuse and altering myosin gene expression of through a mechanism of fiber type control like calcineurin, or activating other pathways involved in oxidative metabolism such as HIFα. It may also be limiting the turnover of cardiolipin as 8 d of denervation in adult rats results in a reduction in cardiolipin content and alterations in key metabolic regulators of cardiolipin metabolism (Ostojić et al., 2013). Whether these gene outcomes have any practical effect needs to be confirmed in future studies as muscle mass was not preserved and there were no other clear effects of SS‐31 in the present study.

Our data shows that a daily 5.0 mg/kg dose of SS‐31 for 14 d did not prevent or slow muscle atrophy following a moderate contusion SCI. There were no changes in protein synthesis, markers of protein carbonylation or gene expression, with the exception of SS‐31‐associated changes in mRNA of *MYH7* and *MYH2*. There are some clear limitations of our study. The animals which received an SCI recovered some locomotor function and we may have missed the window in which SS‐31 may have had a beneficial effect, either on muscle mass or function. Relatedly, we did not measure any type of respiratory or contractile function, outcomes which may demonstrate a practical effect outside of the biochemical and molecular results we present. While SS‐31 has not been associated with neurological recovery in other pre‐clinical pathologies (Petri et al., 2006), it has shown some capacity to protect pulmonary function after SCI (Zhu et al., 2017). Long‐term locomotor recovery after SCI may be an avenue in which SS‐31 could have beneficial effect. Lastly, we did not measure whether SS‐31 has an effect on mitochondrial enzymatic function or ROS production, either in fibers or in isolated mitochondria. This would provide some insight into protective metabolic function that may not be easily observed in our current study.

## CONFLICT OF INTEREST

The authors have no conflicts of interest or financial disclosures to report.

## AUTHOR CONTRIBUTIONS

ZAG and CPC conceived the study and experimental designs. ZAG performed all experiments and data analyses. ZAG, JJD, CPC, and MMB provided reagents and supplies. ZAG wrote the manuscript. ZAG, JJD, CPC, and MMB edited and revised the manuscript. Lastly, all authors approved the final version of the manuscript.
